# Exploring the opportunity for therapeutic drug monitoring (TDM) and precision dose antimicrobials in an outpatient antimicrobial therapy (OPAT) service: a prospective observational study

**DOI:** 10.1093/jac/dkaf484

**Published:** 2026-01-29

**Authors:** R C Wilson, M J Gilchrist, R Cele, S d’Arc, E A Mills, C Ndhlovu, B Balancio, P Arkell, L Read, M Bayliss, A R Noel, A P MacGowan, A H Holmes, T M Rawson

**Affiliations:** Centre for Antimicrobial Optimisation, Imperial College London, London, UK; Pharmacy Department, Imperial College Healthcare NHS Trust, London, UK; National Institute for Health Research Health Protection Research Unit in Healthcare Associated Infections and Antimicrobial Resistance, Imperial College London, London, UK; David Price Evans Infectious Diseases & Global Health Group, The University of Liverpool, Liverpool, UK; Centre for Antimicrobial Optimisation, Imperial College London, London, UK; Pharmacy Department, Imperial College Healthcare NHS Trust, London, UK; National Institute for Health Research Health Protection Research Unit in Healthcare Associated Infections and Antimicrobial Resistance, Imperial College London, London, UK; OPAT Service, Imperial College Healthcare NHS Trust, London, UK; OPAT Service, Imperial College Healthcare NHS Trust, London, UK; Department of Infectious Disease, Imperial College London, Charing Cross Hospital Campus, London, UK; Department of Infectious Disease, Imperial College London, Charing Cross Hospital Campus, London, UK; OPAT Service, Imperial College Healthcare NHS Trust, London, UK; OPAT Service, Imperial College Healthcare NHS Trust, London, UK; Centre for Antimicrobial Optimisation, Imperial College London, London, UK; Antimicrobial Reference Laboratory, Infection Sciences, North Bristol NHS Trust, Bristol, UK; Antimicrobial Reference Laboratory, Infection Sciences, North Bristol NHS Trust, Bristol, UK; Antimicrobial Reference Laboratory, Infection Sciences, North Bristol NHS Trust, Bristol, UK; Antimicrobial Reference Laboratory, Infection Sciences, North Bristol NHS Trust, Bristol, UK; Centre for Antimicrobial Optimisation, Imperial College London, London, UK; National Institute for Health Research Health Protection Research Unit in Healthcare Associated Infections and Antimicrobial Resistance, Imperial College London, London, UK; David Price Evans Infectious Diseases & Global Health Group, The University of Liverpool, Liverpool, UK; Fleming Initiative, Fleming Centre, Imperial College London and Imperial College Healthcare NHS Trust, London, UK; Centre for Antimicrobial Optimisation, Imperial College London, London, UK; National Institute for Health Research Health Protection Research Unit in Healthcare Associated Infections and Antimicrobial Resistance, Imperial College London, London, UK; Fleming Initiative, Fleming Centre, Imperial College London and Imperial College Healthcare NHS Trust, London, UK

## Abstract

**Objectives:**

Notwithstanding the wide uptake of outpatient antimicrobial therapy (OPAT) in the UK, there is a paucity of data on antimicrobial exposure. We aimed to assess antimicrobial pharmacokinetic–pharmacodynamics (PK–PD) for several agents in our service with a view to understanding a potential role for therapeutic drug monitoring (TDM) and precision antimicrobial therapy in this setting.

**Methods:**

The study was a prospective, observational, pilot PK–PD study. Adult patients receiving intravenous ceftazidime, ceftriaxone, daptomycin, ertapenem, flucloxacillin or teicoplanin, or oral treatment with linezolid were enrolled. Peak and trough blood samples were obtained. Total and unbound antibacterial concentrations were measured. Clinical details, laboratory findings and UK OPAT good practice recommendation metrics were extracted from the medical record. PK–PD was interpreted according to EUCAST rationale documents and other published guidance. Doses were not adjusted except for linezolid and teicoplanin.

**Results:**

In total, 39 patients were recruited to the study, predominantly on ceftriaxone (21/39, 54%) or ertapenem (6/39, 15%). Four patients received (10%) teicoplanin, three (8%) ceftazidime, three (8%) flucloxacillin, one (3%) linezolid and one (3%) daptomycin. The most common reason for OPAT was bacteraemia and endocarditis (6/39; 15% each). PK–PD target attainment was acceptable for all drugs and dose regimens, and all β-lactam-based treatments met conservative PK–PD targets.

**Conclusions:**

We have demonstrated that drug monitoring is practicable in the OPAT setting of a large institution with no on-site analytical capability. Current dosage regimens result in acceptable PK–PD target attainment. Our findings provide an initial step towards supporting TDM in OPAT.

## Introduction

Outpatient parenteral antimicrobial therapy (OPAT) involves the delivery of parenteral (intravenous) antimicrobial drugs within a defined clinical management plan to patients who are medically stable and do not require inpatient care. Types of infection that are commonly referred to OPAT are dependent on the local population and speciality case mix, but commonly include skin and soft tissue infections, bronchiectasis, deep-seated infections such as intra-abdominal collections or bone and joint infection, and multi-drug resistant organisms with no oral antimicrobial option.

OPAT can reduce hospital bed occupancy, reduce costs of healthcare and improve patient satisfaction compared with inpatient care.^1,2^ In the last 10 years, OPAT services across the UK have grown exponentially but it has mainly been due to the availability of once daily agents or increasing data on continuous infusions and convenience of delivery.

Despite the widespread adoption of OPAT, data on antimicrobial exposure in this setting remain limited, and antimicrobial pharmacokinetic–pharmacodynamic (PK–PD) target attainment has not been well evaluated for most agents used. Dosing is frequently based on a one size fits all approach.

In 2019, EUCAST updated the ceftriaxone dose used to set the clinical breakpoint for MSSA infections. The revised guideline increased the ceftriaxone dose from 2 g once a day (OD) to either 2 g twice a day (BD) or 4 g OD to achieve acceptable PTA and minimize the risk of treatment failure.^3^ While this change was not a recommendation for institutions to increase their usual ceftriaxone dose many OPAT services chose to. The increased dose presented a potential challenge to the delivery of OPAT given limited safety and efficacy data for higher ceftriaxone doses in this setting, and a greater potential burden on services to administer the additional daily doses. No known PK–PD data are available describing whether a higher ceftriaxone dose is appropriate or necessary within this population.

The optimization of antimicrobial PK–PD in OPAT is particularly important given that longer drug exposures required in this setting are known to be risk factors for toxicity and the selection of drug-resistance, and there are limited data to describe the effect of higher drug exposures for a prolonged period of time.

Therapeutic drug monitoring (TDM) is the process of measuring drug exposure with a view to manipulating the dose to achieve a set target ensuring safety and efficacy. TDM might support antibacterial precision dosing in the OPAT setting where less frequent administration or continuous infusions are the preferred mode of delivery, but where there is relatively less evidence to support such dosing structures. TDM might also help to guide the service in cases where drug stability is of concern, such as for meropenem.^4^ The present application of TDM in OPAT is limited to case series.^5,6^

The first step must be to understand what antimicrobial exposures are currently achieved and their association with outcomes of interest. This pilot study prospectively evaluated the feasibility of measuring drug concentrations and estimating drug exposure for patients receiving routine antimicrobial therapy within our OPAT service.

## Method

### Study design

This study was a prospective observational pilot PK–PD study of outpatients enrolled on the Imperial College Healthcare NHS Trust (London, UK) OPAT service. All antibacterials were prescribed in line with local protocols or antimicrobial susceptibility testing results. Data were collected as part of routine NHS care. Doses were not adjusted based on observed concentrations except for pre-dose teicoplanin and pre-dose linezolid as part of routine clinical practice. The study was locally approved as a service evaluation (registration number 556). Ethical approval was waived in line with local policy.

### Participant recruitment

Male and female patients 18 years and older on the Imperial College Healthcare NHS Trust OPAT service who had received at least 7 days of treatment with intravenous intermittent ceftazidime, ceftriaxone, daptomycin, ertapenem infusions, teicoplanin, continuous flucloxacillin infusions or oral treatment with linezolid were eligible for recruitment. Recruitment took place between March 2021 and April 2022.

### Sample collection and preparation

Blood samples were collected in serum-separating tubes (BD Vacutainer^™^ SSTII^™^) immediately before (pre-dose, timepoint 1, T1) and 30–60 min after (post-dose, timepoint 2, T2) the antibacterial dose was administered during a single OPAT clinic visit. These timepoints were chosen to correspond with the patient’s clinic visit.

### Bioanalysis

Samples containing the β-lactams (ceftazidime, ceftriaxone, ertapenem and flucloxacillin) or daptomycin were left to clot at room temperature and then held on ice until centrifugation at 3000g (4–8°C) for 5 min. Samples were transferred under cold storage and analysed by the Antimicrobial Reference Laboratory (ARL) (Bristol, UK) using high-performance liquid chromatography with tandem mass spectrometry. Both total and unbound concentrations were measured for the β-lactams and daptomycin. Linezolid and teicoplanin samples were processed by the local laboratory and analysed by the ARL. Only total concentration was measured for linezolid and teicoplanin by high-performance liquid chromatography with ultraviolet detection and Quantitative Microsphere System (Quantitative Microsphere System^®^) immunoassay, respectively. The Supplementary File gives further details on the analytical method (see File S1 available as Supplementary data at JAC Online).

### Data collection

OPAT diagnosis and treatment details, biochemistry and haematological parameters were extracted from the electronic medical record. Details on the last antimicrobial dose administered and timing of the observed dose and sampling were recorded prospectively by nurses in the OPAT team.

Bacterial susceptibility was tested by disc diffusion or broth microdilution, and interpreted from the EUCAST breakpoint tables, version 13.1.^7^

### Definitions for adverse events

Reference ranges for alanine transaminase (ALT), alkaline phosphatase (ALP), neutrophils and platelets are defined by the local biochemistry laboratory. Normal ranges were defined as ALT below 34 IU/L, ALP below 130 IU/L, platelets 135–400 × 10^9^/L and neutrophils 2.0–7.1 × 10^9^/L. Clinically significant raised ALT or ALP was defined as three times the upper limit of normal. Clinically significant neutropenia was defined as neutrophils below 1.0 × 10^9^/L and thrombocytopenia as below 50 × 10^9^/L. Patients receiving daptomycin were assessed for elevation in creatinine kinase weekly with the reference range defined as 25–200 IU/L. All biochemical and haematological parameters are routinely measured once weekly as part of the outpatient appointment.

### Statistical analysis and data reporting

Data were analysed in R (v.4.2.1).^8^ Demographics are shown as mean and standard deviation (SD), or median and IQR. Estimated glomerular filtration rate (eGFR) was calculated with the CKD-EPI equation.^9^ Drug levels below the limit of detection (LoD) were excluded from analysis. Clinical diagnoses and outcomes of OPAT are presented in accordance with BSAC good practice recommendations where available (see Tables S1 and S2).^10^ Drug concentrations achieved are presented with univariate analysis, and a coefficient of variation (CV) was calculated for each drug dose. Normality of data were assessed visually and using the Shapiro–Wilk test. Statistical significance between drug dosages for ceftriaxone are compared with the Wilcoxon signed-rank test.

For linezolid and teicoplanin reference ranges from the ARL and previous publications were used to assess individual target attainment.^11,12^

The remaining antibiotic concentrations were analysed *post hoc* and assessed against relevant PK–PD targets. For the β-lactams (excluding flucloxacillin) two PK–PD targets were explored. The first was derived from EUCAST rationale documents (https://www.eucast.org/) as a drug-specific percentage *f*T > MIC, and the second against a higher 100%*f*T > MIC target that has been advocated in clinical scenarios such as sepsis.^13^ For flucloxacillin, only the second target was evaluated as there is no rationale document available. The MIC was defined in order of preference by measured MIC, or if unavailable the EUCAST resistant breakpoint. For the β-lactams, when the observed unbound trough concentration was greater than the MIC then targets were achieved. However, if the unbound trough concentration was below the MIC (i.e. <100% of *f*T > MIC) the exact *f*T > MIC was estimated in ID-ODS^™^ ((Individually Designed Optimum Dosing Strategies) and assessed against the relevant PK–PD target. ID-ODS (https://www.optimum-dosing-strategies.org) is a web application powered by R and wrapped in Shiny. It incorporates an extensive model library alongside Bayesian feedback to estimate individual antibiotic exposure.

For daptomycin, area under the concentration–time curve (AUC, mg.h/L) was estimated for each patient in ID-ODS. Total AUC was estimated for staphylococcal infections and unbound AUC for enterococcal infections.^14^ Total trough level was used for toxicity.^15^

Results are reported in line with the STROBE statement on epidemiological studies.^16^

## Results

In total, 39 patients were recruited to the study. Twenty-one (54%) received ceftriaxone, six (15%) ertapenem, four (10%) teicoplanin, three (8%) ceftazidime, three (8%) flucloxacillin, one (3%) linezolid and one (3%) daptomycin. In addition, two of the 39 patients received daptomycin in combination with ceftriaxone or ertapenem. For these individuals, both antibacterials were measured.

### Patient characteristics

Patient characteristics are shown in Table 1. Most patients were male (22/39; 56%). Mean (SD) age was 62 (15) years and weight was 83 (25) kg. The median (IQR) time spent in OPAT was 31 (21–41) days. At the start of treatment mean (SD) albumin concentration was 33 (5) g/L, median (IQR) alanine transaminase was 20 (10–25) unit/L and creatinine was 66 (59–80) micromoles/L. eGFR was normal (>90 mL/min/1.73 m^2^) in 20/39 (51%) and mildly impaired (60–89 mL/min/1.73 m^2^) in 13/39 (33%) patients. In the OPAT population, 15/39 (38%) patients were treated with more than one antibacterial at the point of sampling. Including the two patients on daptomycin in combination with ceftriaxone or ertapenem, the other combination antibacterials were metronidazole (6/39; 15%) and 1/39 (3%) on each of ciprofloxacin, clindamycin, co-trimoxazole, levofloxacin, linezolid, meropenem and rifampicin.

**Table 1.** Demographics

	Ceftazidime	Ceftriaxone	Daptomycin	Ertapenem	Flucloxacillin	Linezolid	Teicoplanin	Total
Patients, *n* (%)	3 (8)	21 (54)	1 (3)^a^	6 (15)	3 (8)	1 (3)	4 (10)	39 (100)
Male, *n* (%)	2 (5)	12 (31)	0 (0)	4 (10)	1 (3)	1 (3)	2 (5)	22 (56)
Age, mean (SD) years	56 (7)	61 (16)	69 (NA)	69 (12)	65 (27)	36 (NA)	66 (9)	62 (15)
Weight, mean (SD) kg	81 (13)	84 (25)	69 (NA)	88 (35)	67 (15)	86 (NA)	89 (31)	83 (25)
OPAT days, median (IQR) days	41 (35–41)	31 (20–40)	58 (NA)	38 (32–42)	25 (23–28)	20 (NA)	31 (22–50)	31 (21–41)
Full blood count								
White cell count, median (IQR) ×10^9^/L	6.2 (6.1–8)	7.3 (6–8)	7.6 (NA)	6.6 (5.3–8.5)	6.9 (4.7–8.9)	7.2 (NA)	4.0 (3.6–5.1)	6.9 (5.4–8.1)
Neutrophils, mean (SD) ×10^9^/L	4.6 (2.5)	4.2 (1.5)	4.4 (NA)	4.2 (1.5)	5 (3.1)	3.7 (NA)	3.2 (2.4)	4.2 (1.7)
Platelets, mean (SD) ×10^9^/L	336 (126)	273 (63)	211 (NA)	356 (174)	208 (155)	354 (NA)	213 (136)	280 (109)
Biochemistry								
Albumin, mean (SD) g/dL	36 (6)	35 (6)	32 (NA)	30 (4)	28 (5)	35 (NA)	33 (4)	33 (5)
Alanine transaminase, median (IQR) unit/L	36 (26–42)	21 (11–29)	6 (NA)	15 (11–21)	9 (9–10)	24 (NA)	21 (16–37)	20 (10–25)
Creatinine, median (IQR) micromoles/L	57 (57–61)	69 (61–83)	53 (NA)	73 (60–89)	65 (60–91)	63 (NA)	74 (65–118)	66 (59–80)
Estimated GFR, *n* (%) mL/min/1.73 m^2^								
> 90	3 (8)	9 (23)	1 (3)	2 (5)	2 (5)	1 (3)	2 (5)	20 (51)
60–89	0 (0)	8 (21)	0 (0)	4 (10)	0 (0)	0 (0)	1 (3)	13 (33)
45–59	0 (0)	3 (8)	0 (0)	0 (0)	0 (0)	0 (0)	0 (0)	3 (8)
30–44	0 (0)	1 (3)	0 (0)	0 (0)	1 (3)	0 (0)	0 (0)	2 (5)
< 30	0 (0)	0 (0)	0 (0)	0 (0)	0 (0)	0 (0)	1 (3)	1 (3)

^a^1 patient ceftriaxone + daptomycin, 1 patient ertapenem + daptomycin, not included in daptomycin figures.

### Infection characteristics

Infection characteristics are shown in Table 2. The most common reason for OPAT was bacteraemia/blood stream infection/septicaemia (6/39; 15%) and endocarditis (6/39; 15%), followed by discitis/vertebral osteomyelitis (4/39; 10%), empyema (4/39; 10%) and osteomyelitis (4/39; 10%). In total, 15/39 (38%) patients had no significant microbiology available. Antimicrobial susceptibility data were available for 24/39 (62%) patients, of which 14/24 (58%) had an MIC reported and the remaining 10/24 (42%) had sensitivity confirmed by disc diffusion.

**Table 2.** Infection characteristics

Drug	Infection diagnosis	Microbiology	Source	Sensitivities	SIR	MIC^a^	Count
Ceftazidime	Malignant otitis externa	Negative	NA	NA	NA	NA	1
	Respiratory tract infection: other	Negative	NA	NA	NA	NA	1
	Sinusitis	*Pseudomonas aeruginosa*	Pus (superficial site)	Ceftazidime	S	NA	1
Ceftriaxone	Empyema	Negative	NA	NA	NA	NA	2
	Osteomyelitis: non-surgical	Negative	NA	NA	NA	NA	2
	Bacteraemia/blood stream infection/septicaemia	*Staphylococcus aureus* and *Escherichia coli*	Blood culture	Oxacillin/ceftriaxone	S/S	0.75/NA	1
	Bacteraemia/blood stream infection/septicaemia	*Staphylococcus aureus*	Blood culture	Oxacillin	S	0.5	1
	Cellulitis	Negative	NA	NA	NA	NA	1
	Cerebral abscess	Negative	NA	NA	NA	NA	1
	Discitis/vertebral osteomyelitis: no metalwork	Negative	NA	NA	NA	NA	1
	Discitis/vertebral osteomyelitis: no metalwork	*Streptococcus gallolyticus*	Blood culture	Penicillin	S	0.064	1
	Endocarditis	Negative	NA	NA	NA	NA	1
	Endocarditis	Streptococci: beta haemolytic, Group B	Blood culture	Penicillin	S	0.032	1
	Endocarditis	Streptococci: beta haemolytic, Group B	Blood culture	Penicillin	S	NA	1
	Endocarditis	*Streptococcus oralis*	Blood culture	NA	S	NA	1
	Endocarditis	*Streptococcus parasanguinus*	Blood culture	Penicillin	S	0.016	1
	Endocarditis	*Streptococcus sanguis*	Blood culture	Penicillin	S	0.094	1
	Infected pacing box	*Staphylococcus aureus*	Pus (deep site)	Oxacillin	S	0.75	1
	Septic arthritis	Negative	NA	NA	NA	NA	1
	Sinusitis	*Escherichia coli* +++ and *Streptococcus anginosus* +++	Pus (superficial site)	Ceftriaxone	S	NA	1
	Skin and soft tissue infection	*Streptococcus oralis* and *Streptococcus constellatus*	Bone (intraoperative)	Penicillin	S	NA	1
Ceftriaxone and daptomycin	Cerebral abscess	*Staphylococcus aureus*	Pus (deep site)	Oxacillin/daptomycin	S	0.75/0.5	1
Daptomycin	Prosthetic joint, knee	*Staphylococcus epidermidis*	Tissue (Intraoperative)	Daptomycin	S	0.75	1
Ertapenem	Discitis/vertebral osteomyelitis: no metalwork	Negative	NA	NA	NA	NA	2
	Intra-abdominal abscess	*Klebsiella pneumoniae*	Blood culture	Meropenem	S	NA	1
	Osteomyelitis: non-surgical	*Klebsiella variicola*	Blood culture	Co-amoxiclav	S	NA	1
	Skin and soft tissue infection^b^	Staphylococcus aureus	Swab (Wound)	Flucloxacillin	S	NA	1
Ertapenem and daptomycin	Hepatic abscess	*Enterococcus faecalis*, *Escherichia coli* (ESBL) and *Staphylococcus aureus*	Tissue (Biopsy)	Carbapenem/vancomycin	S/S	NA	1
Flucloxacillin	Bacteraemia/blood stream infection/septicaemia	*Staphylococcus aureus*	Blood culture	Oxacillin	S	0.5	1
	Bacteraemia/blood stream infection/septicaemia	*Staphylococcus aureus*	Blood culture	Oxacillin	S	1	1
	Bacteraemia/blood stream infection/septicaemia	*Staphylococcus aureus*	Blood culture	Oxacillin	S	1.5	1
Linezolid	Empyema	*Streptococcus pyogenes*	Pleural fluid	Penicillin	S	NA	1
Teicoplanin	Bacteraemia/blood stream infection/septicaemia	*Enterococcus faecalis*	Blood culture	Teicoplanin	S	0.75	1
	Empyema	Negative	NA	NA	NA	NA	1
	Intra-abdominal abscess	Negative	NA	NA	NA	NA	1
	Osteomyelitis: non-surgical	*Staphylococcus aureus*	Bone (intraoperative)	Teicoplanin	S	NA	1

^a^The SIR (susceptible, susceptible increased exposure, resistant) and MIC is given in relation to the drug in the sensitivities column.

^b^Hidradenitis suppurativa.

### Antibacterial bioanalysis

**Figure 1.**
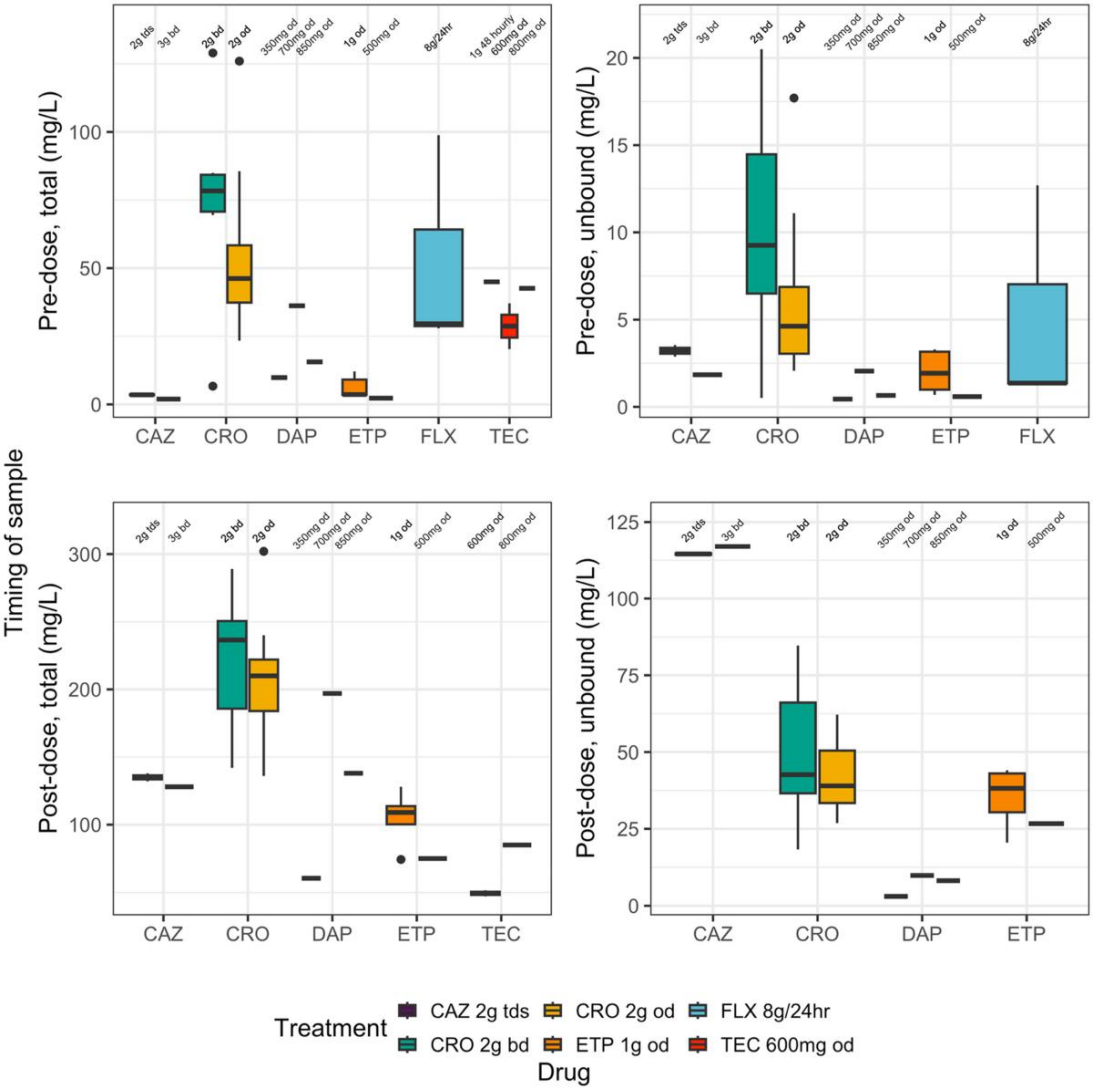
Total and unbound concentrations. CAZ, ceftazidime; CRO, ceftriaxone; DAP, daptomycin; ETP, ertapenem; FLX, flucloxacillin; TEC, teicoplanin. Unbound levels not measured for teicoplanin. Flucloxacillin levels only taken once before (pre-dose) the continuous-infusion pump was changed. Shaded boxes indicate results from more than one patient. Dose regimens are provided across the top of each facet.

Antibacterial concentrations are shown in Figure 1 and Table 3. Most patients (30/39; 77%) were sampled at both timepoints. In 4/39 (10%) participants T2 was unavailable due to logistical difficulties with sampling and in 1/39 (3%) participant T1 was excluded due to missed doses before the clinic visit. One ceftriaxone unbound sample was excluded as below the LoD.

**Table 3.** Drug concentrations and binding characteristics

Drug	Regimen	Count	T1 Total mg/L	CV (%)	T1 Unbound mg/L	CV (%)	T1 Fraction unbound	T2 Total mg/L	CV (%)	T2 Unbound mg/L	CV (%)	T2 Fraction unbound
Ceftazidime	3 g bd	1	1.95 1.95–1.95	NA	1.84 1.84–1.84	NA	0.94 0.94–0.94	128 128–128	NA	117 117–117	NA	0.91 0.91–0.91
	2 g tds	2	3.51 3.36–3.67	12.27	3.21 3.05–3.38	14.74	0.91 0.91–0.92	135 133.5–136.5	3.14	114.5 114.25–114.75	0.62	0.85 0.84–0.86
Ceftriaxone	2 g od	14	46.2 37.35–58.4	51.15	4.62 3.04–6.87	72.88	0.1 0.09–0.13	210 184–222	18.43	39 33.4–50.47	25.52	0.19 0.17–0.22
	2 g bd	7	78.35 70.75–84.3	52.9	9.26 6.5–14.47	69.71	0.11 0.08–0.15	236.5 185.75–250.5	24.63	42.65 36.58–66.12	50.04	0.2 0.18–0.27
Daptomycin	350 mg od	1	9.85 9.85–9.85	NA	0.45 0.45–0.45	NA	0.05 0.05–0.05	60.4 60.4–60.4	NA	3.04 3.04–3.04	NA	0.05 0.05–0.05
	700 mg od	1	36.2 36.2–36.2	NA	2.05 2.05–2.05	NA	0.06 0.06–0.06	197 197–197	NA	9.83 9.83–9.83	NA	0.05 0.05–0.05
	850 mg od	1	15.6 15.6–15.6	NA	0.66 0.66–0.66	NA	0.04 0.04–0.04	138 138–138	NA	8.14 8.14–8.14	NA	0.06 0.06–0.06
Ertapenem	500 mg od	1	2.28 2.28–2.28	NA	0.59 0.59–0.59	NA	0.26 0.26–0.26	75 75–75	NA	26.7 26.7–26.7	NA	0.36 0.36–0.36
	1 g od	5	3.62 3.52–9.09	64.44	1.93 0.99–3.16	59.72	0.26 0.22–0.28	109 100.33–113.75	21.31	38.2 30.4–43.05	30.81	0.33 0.3–0.36
Flucloxacillin	8 g/24hr	3	29.6 28.75–64.2	77.64	1.36 1.35–7.03	127.79	0.05 0.05–0.09	NA	NA	NA	NA	NA
Linezolid	600 mg bd PO	1	1.9 1.9–1.9	NA	NA	NA	NA	NA	NA	NA	NA	NA
Teicoplanin	1 g 48 hourly	1	45 45–45	NA	NA	NA	NA	NA	NA	NA	NA	NA
	600 mg od	2	28.7 24.5–32.9	41.39	NA	NA	NA	49.35 48.23–50.48	6.45	NA	NA	NA
	800 mg od	1	42.6 42.6–42.6	NA	NA	NA	NA	85 85–85	NA	NA	NA	NA

Drug concentrations and percentages are median (IQR); BD, twice daily; OD, once daily; PO, oral; T1, pre-dose; T2, post-dose; TDS, three times daily.

Twenty-one (54%) patients were recruited on ceftriaxone (14 patients 2 g once daily, seven patients 2 g twice daily). The free fraction was comparable between dosages but greater at peak concentrations in keeping with known saturable binding characteristics^17^ (pre-dose 10%, post-dose 20%). The median (IQR) pre-dose unbound concentration was greater in the 2 g twice daily group (9.3 mg/L, 6.5–14.5) versus once daily (4.6 mg/L, 3.0–6.9) (*P* = 0.1, Wilcoxon signed-rank test).

Six patients were recruited on ertapenem (five patients 1 g once daily, one patient 500 mg once daily). The fraction unbound was similar between doses but greater post-dose [median (IQR) 26% (22%–28%) and 33% (30%–36%), respectively].

Three patients were recruited on flucloxacillin 8 g/24 h continuous infusions. The measured unbound concentration was median (IQR) 1.36 (1.35–7.03) mg/L. Flucloxacillin was highly bound with a free fraction (IQR) of 5% (5%–9%). One patient had significantly higher unbound concentrations (12.7 mg/L versus 1.33 and 1.36 mg/L, respectively) and a lower serum albumin (23 g/dL versus 30 and 32 g/dL, respectively).

Three patients were recruited on ceftazidime (two patients 2 g three times daily, one patient 3 g twice daily). The pre-dose unbound concentration was lower for the patient on 3 g twice daily versus 2 g three times daily [1.84 mg/L versus median (range) 3.21 (2.88–3.55) mg/L], and the post-dose was higher [117.00 mg/L versus median (range) 114.5 (114–115) mg/L].

Three patients were recruited on daptomycin (350 mg, target 6 mg/kg, actual 7 mg/kg; 700 mg, target and actual 10 mg/kg; and 850 mg, target 6 mg/kg, actual 7 mg/kg once daily; doses rounded to vial size for OPAT administration). The pre-dose concentration was 9.85–36.20 mg/L (range) and post-dose was 60.4–197 mg/L (range). The fraction unbound was similar between doses and timepoints (range 4%–6%).

Four patients were recruited on teicoplanin (1 g alternate days, target and actual 12 mg/kg; 800 mg once daily, target 12 mg/kg, actual 9 mg/kg; 600 mg once daily, target 12 mg/kg, actual 10 mg/kg; 600 mg once daily, target 6 mg/kg, actual 5 mg/kg; doses adjusted for renal function and rounded to vial size for OPAT administration). Pre-dose levels were 20.3–45.0 mg/L (range). Post-dose levels were only measured for two out of four (50%) patients and were 47.1–85.0 mg/L (range).

One patient was recruited on linezolid 600 mg twice daily and the pre-dose level was 1.9 mg/L. A post-dose level was not taken.

### Outcomes

The treatment aim was cure in 38/39 (97%) patients and improvement in one patient out of 39 (3%). The treatment aim was attained in 38/39 (97%) patients. For one patient out of 39 (3%), their outcome was not attained following hospital readmission for complications of their infection.

### Individual PK–PD target attainment

Target attainment is shown in Table 4. PK–PD parameters were individually estimated in ID-ODS for 7/41 (17%) drug administrations. For the β-lactams, all patients met the conservative target, with most meeting the higher target, except for ceftazidime and one patient on ceftriaxone.

**Table 4.** Target attainment

Drug	Regimen	*n* (patients)	*n* (samples T1/T2)	Assay	Target 1	Achieved	Target 2	Achieved	MIC	Estimated in ID-ODS
Ceftazidime	3 g bd	1	1/1	HPLC-MS/MS	65%>*f*TMIC^18^	1	100%*f*T > MIC	0	8^7^	1
	2 g tds	2	2/2	HPLC-MS/MS	65%>*f*TMIC^18^	2	100%*f*T > MIC	0	8^7^	2
	Subtotal	3	3/3	.	.	3	.	0	.	3
Ceftriaxone	2 g od	14	14/13	HPLC-MS/MS	30%>*f*TMIC^19^	14	100%*f*T > MIC	14	2^19^	0
	2 g bd	6	6/6	HPLC-MS/MS	30%>*f*TMIC^19^	6	100%*f*T > MIC	5	2^19^	1
	Excluded	1	0/0		30%>*f*TMIC^19^	0	100%*f*T > MIC	0	2^19^	0
	Subtotal	20	20/19			20	.	19	.	1
Daptomycin	350 mg od	1	1/1	HPLC-MS/MS	Total AUC/MIC > 666–1061^14^	1	Total trough < 24.3 mg/L	1	0.5^7^	1
	700 mg od	1	1/1	HPLC-MS/MS	Free AUC/MIC > 27.4^14^	1	Total trough < 24.3 mg/L	0	1^14^	1
	850 mg od	1	1/1	HPLC-MS/MS	Total AUC/MIC > 666–1061^14^	1	Total trough < 24.3 mg/L	1	0.5^7^	1
	Subtotal	3	3/3	.	.	3	.	2	.	3
Ertapenem	500 mg od	1	1/1	HPLC-MS/MS	55%>*f*TMIC^20^	1	100%*f*T > MIC	1	0.5^7^	0
	1 g od	5	5/4	HPLC-MS/MS	55%>*f*TMIC^20^	5	100%*f*T > MIC	5	0.5^7^	0
	Subtotal	6	6/5	.	.	6	.	6	.	0
Flucloxacillin	8 g/24hr	3	3/0	HPLC-MS/MS	100%>*f*TMIC	3	.	.	0.5–1.5^a^	0
Linezolid	600 mg bd PO	1	1/0	HPLC-UV	Trough >2 mg/L^12^	0	.	.	.	0
Teicoplanin	1 g 48 hourly	1	1/0	QMS Immunoassay	Trough 20–40 mg/L^11^	0	Peak 40–100 mg/L^11^	Missing	.	0
	600 mg od	2	2/2	QMS Immunoassay	Trough 20–40 mg/L^11^	2	Peak 40–100 mg/L^11^	2	.	0
	800 mg od	1	1/1	QMS Immunoassay	Trough 20–40 mg/L^11^	0	Peak 40–100 mg/L^11^	1	.	0
	Subtotal	4	4/3	.	.	2		3	.	0

^a^Observed MIC. BD, twice daily; HPLC-MS/MS, high-performance liquid chromatography with tandem mass spectrometry; HPLC-UV, high-performance liquid chromatography with ultraviolet detection; OD, once daily; QMS, Quantitative Microsphere System; T1, timepoint 1; T2, timepoint 2; TDS, three times daily.

Both AUC-based and trough level-based targets were attained in two out of three (66%) patients on daptomycin, with the remaining patient having a high trough level.

For the patients on teicoplanin, two of the pre-dose levels were supratherapeutic. One of those patients had renal impairment and the same dose was continued with repeat monitoring, and the other patient completed therapy a few days after the level was reported. The dose was not changed for the patient on linezolid as it was only marginally subtherapeutic and a post-dose level was unintentionally omitted hindering interpretation.

### Adverse reactions

A summary of adverse events is provided in the supplementary file (see Table S3). Briefly, 34/39 (87%) patients experienced an adverse drug event comprising 58 adverse drug events in total. Of those adverse drug events only three (3/58, 5%) were clinically significant and considered unrelated to antimicrobial treatment (preexisting fatty liver one out of three, 33%; leukaemia two out of three, 66%). Mild derangement in ALT and ALP was common (18/39, 46% patients). There were no derangements in creatinine kinase for patients treated with daptomycin.

## Discussion

We present drug exposure data for a representative cohort of patients receiving antibacterial treatment via OPAT in the UK.^2^ A diverse range of infections are included. Clinical outcomes recorded as per the BSAC good practice recommendations were attained, with only one outcome not attained. Most patients had either normal or mildly impaired renal function, and mean albumin concentrations (33 g/dL) were slightly below our local reference range (35–50 g/dL), reflecting a population being managed in the outpatient setting.

Most patients included received either ceftriaxone or ertapenem. Peak total ceftriaxone concentrations after 1 g bolus doses have been reported on average 199 mg/L,^21^ which is similar to the median reported in this study for both 2 g once daily and 2 g twice daily when given as a 30-min infusion (210 and 237 mg/L, respectively). Pre-dose ceftriaxone free fraction was approximately double that referenced in the Summary of Product Characteristics^17^ (5% versus 10% in our study), and the absolute difference was maintained at higher concentrations (15% versus 20%, respectively). The difference in median pre-dose concentrations between 2 g once daily and 2 g twice daily was approximately double but not statistically significant. However, our study was not powered to detect this difference. Unbound ceftriaxone concentrations at both timepoints varied widely with a pre-dose CV ∼70% for both doses and post-dose CV ∼25%–50% for 2 g once daily and 2 g twice daily, respectively. Total ceftriaxone variability was lower (pre-dose CV ∼50% for both doses, and post-dose CV ∼18%–25% for 2 g once daily and 2 g twice daily, respectively). Roberts *et al*. reported considerably wider variation in β-lactam concentrations in critical care patients,^22^ however, comparable data outside of this population are unavailable. PK–PD target attainment was good with both conservative and higher targets satisfied in most patients irrespective of dosing regimen.

The association between ceftriaxone and neutropenia is well documented.^23,24^ While a possible signal was observed between total pre-dose concentration and neutropenia in the 2 g once daily group, this was not consistent with the higher concentrations in the 2 g twice daily group. This finding might be confounded by the imbalanced dataset because twice the number of patients received 2 g once daily versus twice daily dosing.

Ertapenem binding was lower in our study than in healthy volunteers. Majumdar *et al*.^25^ report ∼8% unbound at total concentrations <150 mg/L, whereas we observed 26%–33%. Our analytical methodology (provided in detail for ertapenem in Supplementary File section 4), and results, are closer to those by Liebchen *et al*.^26^ who reported median (range) 37.8% (30.9%–53.6%) in an ICU population. Liebchen *et al*.^26^ also noted analytical differences in unbound concentrations depending on the application of physiological conditions (temperature, pH) and ultrafiltration speed. Median maximum total concentrations were lower than expected in our study compared with Majumdar *et al*.^25^ (109.0 mg/L versus 145–175 mg/L, respectively) and median minimum concentrations were higher (3.62 versus 1.2–1.9 mg/L, respectively). The study by Majumdar *et al*.^25^ was single dose, however, the amount of drug and infusion duration are the same. Our ertapenem population had lower albumin levels (mean 30 g/dL), which might have increased the volume of distribution and clearance compared with healthy volunteers, resulting in the difference in observed concentrations.

The unbound flucloxacillin concentrations observed for patients on continuous infusion are reassuring and correspond to 100%*f*T > MIC if integrated against the individual microorganism’s MIC. Our observed total flucloxacillin concentrations were lower than reported in other OPAT patients,^27^ but within those seen in ICU.^28^

Notwithstanding higher ceftazidime doses, our total trough concentrations were lower than early data that showed minimum trough concentrations of 5.6 mg/L for a lower 1.5 g three times daily dose in surgical ICU patients.^29^ Data predicting toxicity based on drug concentrations are not available for ceftazidime, although one study used 100 mg/L based on results from a similar cephalosporin, cefepime.^30^ Neurotoxicity or any other significant toxicity was not observed in our ceftazidime group despite peak concentrations above this. Mean peak unbound concentrations have been previously reported 21.0%^31^ which is higher than observed in our study. While the number of patients recruited on ceftazidime was small we observed the least variability in concentrations in this group.

Daptomycin concentrations were within those previously studied in critical and non-critically ill patients.^32^ There was very little variability in protein binding although our fraction unbound is lower than previous reports in healthy volunteers.^31,33^ We did not observe elevations in creatinine kinase, and only one patient had a trough concentration greater than a previously reported breakpoint predicting toxicity (24.3 mg/L).^15^

Teicoplanin is a well-established agent in the OPAT setting.^2^ For a 12 mg/kg-based regimen trough concentration targets for efficacy (>20 mg/L depending on indication) and peak concentrations (∼100 mg/L) are reported in the Summary of Product Characteristics.^34^ Trough levels >40 mg/L have been associated with a risk of thrombocytopenia.^11^ In our study the median observed trough concentration was ∼40 mg/L suggesting suitable exposure. We did not observe thrombocytopenia. Owing to vial size rounding, our weight-based doses were all below 12 mg/kg and this might have driven the lower peak concentrations.

We only included one patient on linezolid whose trough level was narrowly below target. A level between 2 and 8 mg/L is normally considered efficacious and safe for non-tuberculous indications, although lower upper limits have been proposed for patients on long-term treatment for tuberculosis.^12^

Across the population mild adverse drug reactions, predominantly related to liver injury, were common. However, there were no clinically significant adverse drug reactions related to treatment, adding to existing literature supporting the safety of the OPAT service.^2^ The limited sample size of our dataset precludes any robust comparison of safety profiles.

The study has several limitations. We chose to look at pre- and post-dose levels to ensure minimal impact on patients’ appointment times. However, we acknowledge a general lack of data or guidance in terms of acceptable targets for peak and trough levels for the β-lactams. From a logistical point of view 100%*f*T > MIC is readily evaluated as a binary outcome from a trough level once an MIC has been chosen. If a trough level does not meet the higher target, then it is possible to estimate the percentage *f*T > MIC using Bayesian forecasting software to evaluate whether a conservative target is attained. This approach could be followed in clinical practice with increased access to appropriate software, and additional resource to support sampling, analysis and the interpretation of levels. Other challenges that we encountered included a lack of MIC testing for many isolates and therefore a reliance on resistance breakpoint MIC values, which might underestimate actual target attainment. The study is also limited in that dose adjustments were not made to drug regimens outside of linezolid and teicoplanin in line with current clinical practice.

During our study, we accepted logistical challenges undertaking TDM outside of a specialist centre with on-site capability. Turnaround times become significant if the sample must travel, which if adopted in routine clinical practice, can result in a delay to decision making. Moving away from a decentralized model requires investment because of the accreditation, upkeep and training costs to maintain clinically accredited assays. Conversely, while on-site analysis of drug levels to implement same day dose adjustments in, e.g. the critical care setting, is favourable, there are no data to inform an appropriate reporting window for OPAT. Patient clinical stability and prolonged treatment durations might allow off site testing as used in our study. Other barriers to the implementation of TDM have been discussed in detail elsewhere.^6,35,36^

Our PK samples reflect levels on one day of treatment over a course duration which in some cases continued for many weeks. We are unable to assess the effect of pharmacokinetic variability within the individual over time. Furthermore, the impact of drug exposure on antimicrobial resistance was not explored in this study.

The OPAT population is self-selected to include patients who are most likely to benefit from treatment with a relatively low risk of medical deterioration. The sample is therefore biased towards treatment success, and it is difficult to draw conclusions between the effect of drug exposure and clinical outcome. Any association between drug exposure and clinical outcome would also be confounded by the number of patients on multiple antimicrobials (38%).

We were able to recruit a reasonable number of patients on ceftriaxone and ertapenem, and future work will look more closely at ceftazidime, daptomycin, flucloxacillin, linezolid and teicoplanin, which was unfortunately limited in our sample due to the low frequency of use.

### Conclusion

The study represents our first look at understanding antibacterial drug exposure in the OPAT setting, including both total and unbound antibacterial concentrations. We have demonstrated that drug monitoring as a step towards precision dosing can be deployed as part of OPAT. In our limited sample, drug exposures resulting from current dosing regimens provide acceptable PK–PD target attainment. In line with the UK National Action Plan^37^ there is a need to ensure antimicrobial dosing is appropriate, and manage potential adverse events, but this must be balanced against the logistical requirements of OPAT administration. TDM and precision dosing might allow us to manage more complex infections in the OPAT setting, allow us to use more novel administration delivery options e.g. continuous infusion, and in other cases support earlier IV to oral switching.

## Supplementary Material

dkaf484_Supplementary_Data

## Transparency declarations

T.M.R. received honoraria for consultancy from Sandoz (2020), honoraria for the delivery of educational activities from BioMerieux (2021–2022) and speakers fees from Roche Diagnostics Ltd (2021). M.G. has received consulting fees from Pfizer, Menarini and Napp. A.P.M. has received research grants from Oxford Drug Design, Merck, BioVersyis, Roche, InfectoPharm, iFAST and IMI. He has acted as a consultant/advisor for GSK, BioVersys, Roche, Bicycle Therapeutics and the Ineos Oxford Institute for Antimicrobial Research. All other authors have no potential conflicts of interest to declare.
