# Supplementary material for: Exploring the opportunity for therapeutic drug monitoring (TDM) and precision dose antimicrobials in an outpatient antimicrobial therapy (OPAT) service: a prospective observational study
Source: J Antimicrob Chemother. 2026 Jan 29;81(2):dkaf484. doi: 10.1093/jac/dkaf484 (PMC12853871; doi:10.1093/jac/dkaf484)
Supplement: dkaf484_Supplementary_Data [file dkaf484_supplementary_data.docx]

# Supplementary

1.0 Ceftazidime bioanalysis

1.1 Chemicals and standards

Potassium phosphate, monobasic and dibasic, of Reagent Plus® grade and LCMS grade formic acid; was obtained from Sigma-Aldrich. Ammonium formate of LC/MS quality was obtained from Sigma Aldrich (St. Louis, MO, USA). LC/MS-grade acetonitrile and water was purchased from Fisher Scientific (Loughborough, UK). Fresh frozen serum was purchased from TCS biosciences (Botolph Claydon, UK). Ceftazidime (CAZ) and Ceftriaxone (CRO) (used as an internal standard) were purchased from Sigma Aldrich.

1.2 HPLC-MS/MS conditions and procedure

A Shimadzu Prominence® (Kyoto, Japan) LC system was coupled to a Sciex triple quadrupole 4000 QTRAP mass spectrometer (AB Sciex, Warrington, UK). Sciex Analyst v.1.5.1 software was used to control the HPLC-MS/MS system and collect the MS data. High purity nitrogen was supplied to the MS by a Thyster-TF/1 generator (F-DGSi, Every, France). The LC system consisted of a SIL20AC autosampler, two LC-20AD pumps, a CTO-20 AC column oven and a CBM-20A controller. The autosampler was set a temperature of 4 °C and programmed to inject 5μL of each sample. The mobile phase consisted of 2 mM ammonium formate in water (solution A) and acetonitrile (solution B). A constant flow rate of 0.5 mL/min was used and a step-wise gradient elution was started with 98% eluent A for 0.1 min, followed by 95% eluent B for 5.2 min, and ended with 95% eluent A for 5.6 min, for a total run time of 7 min. Separation was achieved on a Halo C18 (Advanced Materials Technology ®) column (2.7µM, 50X2.1mm ID, Part no. 92812-402). The column oven temperature was set at 40°C. Multiple Reaction Monitoring (MRM) data were collected by the MS using a Turbo V™ source in electrospray ionisation (ESI) positive mode. The source temperature was set at 500 °C, ion spray voltage at 5500 V, ion source gases 1 and 2 at 30 and 40, curtain gas (CUR) at 40 and collision gas (CAD) at 7. Dwell times were set to 30ms. The specific MRM parameters for the analyte and the IS are given below:

| Analyte | Type | Q1 | Q3 | Collision Energy (CE) |
| --- | --- | --- | --- | --- |
| CAZ | Quantification | 547.21 | 468.04 | 12 |
|  | Qualification |  | 396.04 | 22 |
| CRO | Quantification | 555 | 396.1 | 20 |

1.3 Sample preparation and processing

Before injection into the HPLC system, 20μL of sample was dispensed into a 0.5 mL polypropylene Eppendorf® tube. The sample was then mixed thoroughly with 20μL of water and 40μL of CRO (4mg/L) spiked acetonitrile subsequently added and mixed. Patient samples were thawed and vortexed shortly before analysis and processed in the same manner. After being left to stand at ca. 4 °C for 10 min, the sample was centrifuged for 10 min at 17,000 g. 50μL of supernatant was then transferred to a glass HPLC vial containing 450μL of water. The final extract was thoroughly mixed prior to analysis. Then, 5µL of this sample was injected and quantified as described in Section 1.2.

1.4 Plasma protein binding

10 µL of 3M pH 7.4 buffer potassium phosphate buffer was added to 320 µL of serum sample to give a buffer concentration of ca. 0.1M at a pH of 7.40. The buffered sample was incubated at 37°C for 30 minutes and the total CAZ concentration quantified as described in sections 1.2 and 1.3.

Unbound CAZ was measured by pipetting 250 µL of the serum to be analysed into a Centrifree® Ultrafiltration Device (Merck Millipore, Watford, UK). After centrifugation (1500 g, 20 min, 37°C), 20 μL of each filtrate was dispensed into a 0.5 mL polypropylene Eppendorf® tube and 20 μL of blank serum added, followed by mixing. This sample was then extracted and analysed as described in sections 1.2 and 1.3 CAZ concentrations were calculated using calibrators prepared in serum.

2.0 Ceftriaxone bioanalysis

2.1 Chemicals and standards

Ammonium formate of LC/MS quality was obtained from Sigma Aldrich (St. Louis, MO, USA). LC/MS-grade acetonitrile and water was purchased from Fisher Scientific (Loughborough, UK). Fresh frozen serum was purchased from TCS biosciences (Botolph Claydon, UK). Ceftriaxone and Cefotaxime (used as an internal standard) were purchased from Sigma Aldrich.

2.2 HPLC-MS/MS conditions and procedure

A Shimadzu Prominence® (Kyoto, Japan) LC system was coupled to a Sciex triple quadrupole 4000 QTRAP mass spectrometer (AB Sciex, Warrington, UK). Sciex Analyst v.1.5.1 software was used to control the HPLC-MS/MS system and collect the MS data. High purity nitrogen was supplied to the MS by a Thyster-TF/1 generator (F-DGSi, Every, France). The LC system consisted of a SIL20AC autosampler, two LC-20AD pumps, a CTO-20 AC column oven and a CBM-20A controller. The autosampler was set a temperature of 4 °C and programmed to inject 2 μL of each sample. The mobile phase consisted of 2 mM ammonium formate in water (solution A) and acetonitrile (solution B). A constant flow rate of 0.5 mL/min was used and a step-wise gradient elution was started with 98% eluent A for 0.1 min, followed by 95% eluent B for 2.1 min, and ended with 95% eluent A for 2.6 min, for a total run time of 4 min. Separation was achieved on a Halo C18 (Advanced Materials Technology ®) column (2.7µM, 50X2.1mm ID, Part no. 92812-402). The column oven temperature was set at 40°C. Multiple Reaction Monitoring (MRM) data were collected by the MS using a Turbo V™ source in electrospray ionisation (ESI) positive mode. The source temperature was set at 500 °C, ion spray voltage at 5500 V, ion source gases 1 and 2 at 30 and 45, curtain gas (CUR) at 40 and collision gas (CAD) at 7. The MRM transitions for Ceftriaxone and Cefotaxime (IS) were set to m/z 555→396.1 and 456→396 respectively, with a dwell time of 60ms.

2.3 Sample preparation and processing

Before injection into the HPLC system, 20μL of sample was dispensed into a 0.5 mL polypropylene Eppendorf® tube. The sample was then mixed thoroughly with 20μL of water and 40μL of Cefotaxime (4mg/L) spiked acetonitrile subsequently added and mixed. Patient samples were thawed and vortexed shortly before analysis and processed in the same manner. After being left to stand at ca. 4 °C for 10 min, the sample was centrifuged for 15 min at 17,000 g. 50μL of supernatant was then transferred to a glass HPLC vial containing 450μL of water. The final extract was thoroughly mixed prior to analysis. Then, 2µL of this sample was injected and quantified as described in Section 2.2.

2.4 Plasma protein binding

Prior to preparation samples were buffered with 3 mol­^-1^ Sodium phosphate buffer and incubated at 37°C for 30 minutes. Total Ceftriaxone concentration was quantified as described in sections 2.2 and 2.3. Subsequently, unbound Ceftriaxone was quantified by pipetting 0.25mL of the serum to be analyzed into a Centrifree® Ultrafiltration Device (Merck Millipore). After centrifugation at 1500 g for 20 min at 37°C. 20μL of filtrate was dispensed into a 0.5 mL polypropylene Eppendorf® tube. The sample was then mixed thoroughly with 20μL of blank serum and 40μL of Cefotaxime (4mg/L) spiked acetonitrile subsequently added and mixed. After being left to stand at ca. 4 °C for 10 min, the sample was centrifuged for 15 min at 17,000 g. 50μL of supernatant was then transferred to a glass HPLC vial containing 450μL of water. The final extract was thoroughly mixed prior to analysis. Then, 2µL of this sample was injected and quantified as described in Section 2.2.

3.0 Daptomycin bioanalysis

3.1 Chemicals and standards

Potassium phosphate, monobasic and dibasic, of Reagent Plus® grade and LCMS grade formic acid was obtained from Sigma-Aldrich. LC/MS-grade acetonitrile and water was purchased from Fisher Scientific (Loughborough, UK). Fresh frozen serum was purchased from TCS biosciences (Botolph Claydon, UK). Daptomycin (DAP) was purchased from Sigma Aldrich, and Azithromycin (AZM) (used as an internal standard) from Carbosynth.

3.2 HPLC-MS/MS conditions and procedure

A Shimadzu Prominence® (Kyoto, Japan) LC system was coupled to a Sciex triple quadrupole 4000 QTRAP mass spectrometer (AB Sciex, Warrington, UK). Sciex Analyst v.1.5.1 software was used to control the HPLC-MS/MS system and collect the MS data. High purity nitrogen was supplied to the MS by a Thyster-TF/1 generator (F-DGSi, Every, France). The LC system consisted of a SIL20AC autosampler, two LC-20AD pumps, a CTO-20 AC column oven and a CBM-20A controller. The autosampler was set a temperature of 4 °C and programmed to inject 2 μL of each sample. The mobile phase consisted of 0.1% formic acid in water (solution A) and 0.1% formic acid in acetonitrile (solution B). A constant flow rate of 0.5 mL/min was used and a step-wise gradient elution was started with 98% eluent A for 0.1 min, followed immediately by 40% eluent B for 0.5 min, then increasing to 95% eluent B for 0.5 to 1.0 min, holding at 95% eluent B between 1.0 and 1.5 mins then re-equlibrating at 98% eluent between 1.6 and 3.0 mins. Separation was achieved on a 2.6 µm Kinetex™ XBC18 column (50 x 2.1mm ID) purchased from Phenomenex (Macclesfield, UK). The column was protected by an Upchurch filter unit (Hichrom, Reading, UK) fitted with a 0.5 µm fit. The column oven temperature was set at 40°C. Multiple Reaction Monitoring (MRM) data were collected by the MS using a Turbo V™ source in electrospray ionisation (ESI) positive mode. The source temperature was set at 600 °C, ion spray voltage at 5500 V, ion source gases 1 and 2 at 30 and 40, curtain gas (CUR) at 40 and collision gas (CAD) at 7. Dwell times were set to 30ms and 20ms for DAP and AZM respectively. The specific MRM parameters for the analyte and the IS are given below:

| Analyte | Type | Q1 | Q3 | Collision Energy (CE) |
| --- | --- | --- | --- | --- |
| DAP | Quantification | 811.2 | 313.1 | 47 |
|  | Qualification |  | 341.2 | 32 |
| AZM | Quantification | 749.5 | 591.5 | 30 |

3.3 Sample preparation and processing

Before injection into the HPLC system, 20μL of sample was dispensed into a 0.5 mL polypropylene Eppendorf® tube. The sample was then mixed thoroughly with 20μL of 10% ACN and 40μL of AZM (1mg/L) spiked acetonitrile subsequently added and mixed. Patient samples were thawed and vortexed shortly before analysis and processed in the same manner. After being left to stand at ca. 4 °C for 10 min, the sample was centrifuged for 10 min at 17,000 g. 50μL of supernatant was then transferred to a glass HPLC vial containing 50μL of water. The final extract was thoroughly mixed prior to analysis. Then, 2µL of this sample was injected and quantified as described in Section 3.2.

3.4 Plasma protein binding

10 µL of 3M pH 7.4 buffer potassium phosphate buffer was added to 320 µL of serum sample to give a buffer concentration of ca. 0.1M at a pH of 7.40. The buffered sample was incubated at 37°C for 30 minutes and the total DAP concentration quantified as described in sections 3.2 and 3.3.

Unbound DAP was measured by pipetting 250 µL of the serum to be analysed into a Centrifree® Ultrafiltration Device (Merck Millipore, Watford, UK). After centrifugation (1500 g, 20 min, 37°C), 20 μL of each filtrate was dispensed into a 0.5 mL polypropylene Eppendorf® tube and 20 μL of blank serum added, followed by mixing. This sample was then extracted and analysed as described in sections 3.2 and 3.3 DAP concentrations were calculated using calibrators prepared in serum.

4.0 Ertapenem bioanalysis

4.1 Chemicals and standards

Potassium phosphate, monobasic and dibasic, of Reagent Plus® grade, Ertapenem (ETP) and Meropenem (internal standard, IS) were obtained from Sigma Aldrich (Gillingham, UK). LC/MS-grade acetonitrile and water were purchased from Fisher Scientific (Loughborough, UK), blank serum from TCS biosciences (Botolph Claydon, UK).

4.2 HPLC-MS/MS conditions and procedure

The autosampler was set to a temperature of 5 °C and programmed to inject 5 μL of each extracted sample. The mobile phase consisted of 0.1% formic acid in water (eluent A) and 0.1% formic acid in acetonitrile (eluent B), at a flow rate of 0.5 mL/min The gradient elution programme was as follows: 40% B from 0 to 0.5 min, 95% B from 1.0 to 1.5 min, 2% B from 1.6 min to 3.5 min, and finally 40% B at 3.6 min, with a total run time of 4 min. Separation was achieved on a 2.6 µm Kinetex™ XBC18 column (50 x 2.1mm ID) purchased from Phenomenex (Macclesfield, UK). The column was protected by an Upchurch filter unit (Hichrom, Reading, UK) fitted with a 0.5 µm fit. The column oven temperature was set at 40°C. Multiple Reaction Monitoring (MRM) data were collected using a 4000 Q-Trap (AB Sciex, Warrington, UK) equipped with a Turbo V™ source that was operated in electrospray ionisation (ESI) positive mode. The source temperature was set at 550 °C, ion spray voltage at 5500 V, ion source gases 1 and 2 at 30 and 40, curtain gas (CUR) at 40 and collision gas (CAD) at 7 (medium). Declustering Potential (DP), Entrance Potential (EP) and Collision Exit Cell Potential (CXP) were set at 60,10 and 10 respectively. Dwell times were set to 20 mS. The specific MRM parameters for the analyte and the IS are given below:

| Analyte | Type | Q1 | Q3 | Collision Energy (CE) |
| --- | --- | --- | --- | --- |
| ETP | Quantification | 476.1 | 432.1 | 10 |
|  | Qualification |  | 390.1 | 20 |
| IS | Quantification | 384.2 | 141.0 | 15 |
|  | Qualification |  | 254 & 68.1 | 15 & 30 |

4.3 Sample preparation and processing

20 μL aliquots of sample (calibrators, QCs and blanks) were dispensed into 0.5 mL polypropylene Eppendorf® tubes. Each sample was then mixed thoroughly with 20 μL of water. 40 μL of acetonitrile containing 5 mg/L IS was then added and the contents of the tube thoroughly mixed. Clinical samples were thawed, briefly vortexed, then processed in the same manner. After being left to stand at ca. 4 °C for 10 min, the deproteinised samples were centrifuged for (15 min, 17 000 g). 50 μL of supernatant was then transferred to a glass HPLC vial containing 450 μL of water. The final extract was thoroughly mixed prior to analysis by LC-MS as described in Section 4.2.

4.4 Plasma protein binding

10 µL of 3M pH 7.4 buffer potassium phosphate buffer was added to 320 µL of serum sample to give a buffer concentration of ca. 0.1M at a pH of 7.40. The buffered sample was incubated at 37°C for 30 minutes and the total ETP concentration quantified as described in sections 4.2 and 4.3.

Unbound ETP was measured by pipetting 250 µL of the serum to be analysed into a Centrifree® Ultrafiltration Device (Merck Millipore, Watford, UK). After centrifugation (1500 g, 20 min, 37°C), 20 μL of each filtrate was dispensed into a 0.5 mL polypropylene Eppendorf® tube and 20 μL of blank serum added, followed by mixing. This sample was then extracted and analysed as described in sections 4.2 and 4.3 ETP concentrations were calculated using calibrators prepared in serum.

4.5 Validation

**Recoveries of ERT from phosphate buffered serum vs unbuffered serum**

Blank serum was spiked at nominal ERT concentrations of 0.3 (LLOW), 3 (LOW), 30 (MID) and 150 HIGH) mg/L and analysed after the addition of phosphate buffer, or the same volume of water (unbuffered).

Acceptance Criteria:

Measured concentrations for buffered and unbuffered to be within +/-15% of each other.

| QC Level | % Difference (buffered vs.unbuffereed) |
| --- | --- |
| LLOW | 4.1% |
| LOW | 14.9% |
| MID | 7.5% |
| HIGH | 2.7% |

Results and conclusions: Acceptance criteria met.

**Replacement of water with ultrafiltrate in serum assay**

Serum blanks and double blanks were analysed after dilution with either water, as per the normal assay, or ultrafiltrate.

Acceptance Criteria:

MS response of ETP expected be <20% of the LLOQ peak area in the blank.

MS response for the IS in the double blank to be <5% of the response in the blank.

| Parameter/Sample | MS Response | |
| --- | --- | --- |
|  | ERT | IS |
| LLOQ | 1090 | - |
| 20% LLOQ | 218 | - |
| IS | - | 73900 |
| 5% IS | - | 3695 |
| Blank | 287 | 67100 |
| Double Blank | 15 | 13.3 |
| Ultrafiltrate Blank | 285 | 68800 |
| Ultrafiltrate Double Blank | 9.99 | 20.1 |

Results and conclusions: Acceptance criteria met.

Serum and ultrafiltrate blanks and double blanks gave similar MS responses for ERT. ERT responses in the blank samples were marginally greater than 20% with respect to the LLOQ response. IS response in the double blanks was less than <5% MS of that in the blanks.

**Thermal stability of ERT during incubation and recoveries from ultrafiltration**

Blank serum was spiked at nominal ERT concentrations of 0.3 (LLOW), 3 (LOW), 30 (MID) and 150 (HIGH) mg/L. Total and ultrafiltrate concentrations of ERT were then measured in buffered and unbuffered samples for all QCs, after incubation at 37°C, for the following times: 0 (baseline or T0), 30 (T30) and 60 (T60) minutes.

Acceptance Criteria:

% losses from total concentration and unbound to be <15%.

% unbound not to differ by >10% between incubations at 30 and 60 minutes.

Unbound concentrations to be greater in buffered compared to unbuffered samples^1^.

| **ERT Concentrations (mg/L) in buffered samples** | | | | | | |
| --- | --- | --- | --- | --- | --- | --- |
| QC  Level |  | Time Point (mins) | | | % Loss  (T0-T60) | % Difference (T30-T60) |
|  |  | 0 | 30 | 60 |  |  |
| LLOW | Total | 0.332 | 0.336 | 0.243 | 27.0% |  |
|  | Unbound | 0.0367 | 0.0310 | 0.0359 | 2.2% | -16%* |
|  | % Unbound | 11.1% | 9.2% | 14.8% |  |  |
| LOW | Total | 3.44 | 3.31 | 3.40 | 1.3% |  |
|  | Unbound | 0.360 | 0.347 | 0.323 | 10.2% | 7% |
|  | % Unbound | 10.5% | 10.5% | 9.5% |  |  |
| MID | Total | 33.5 | 34.8 | 32.8 | 2.0% |  |
|  | Unbound | 3.49 | 3.28 | 3.39 | 2.7% | -3% |
|  | % Unbound | 10.4% | 9.4% | 10.3% |  |  |
| HIGH | Total | 130 | 140 | 127 | 1.8% |  |
|  | Unbound | 31.1 | 28.6 | 27.4 | 11.8% | 4% |
|  | % Unbound | 24.0% | 20.4% | 21.5% |  |  |

| **ERT Concentrations (mg/L) in unbuffered samples** | | | | | | |
| --- | --- | --- | --- | --- | --- | --- |
| QC  Level |  | Time Point (mins) | | | % Loss  (T0-T60) | % Difference (T30-T60) |
|  |  | 0 | 30 | 60 |  |  |
| LLOW | Total | 0.322 | 0.321 | 0.334 | -3.7% |  |
|  | Unbound | 0.0310 | 0.0309 | 0.0333 | -7.4% | -8%* |
|  | % Unbound | 9.6% | 9.6% | 10.0% |  |  |
| LOW | Total | 3.33 | 3.05 | 3.20 | 4.1% |  |
|  | Unbound | 0.240 | 0.267 | 0.256 | -6.8% | 4% |
|  | % Unbound | 7.2% | 8.8% | 8.0% |  |  |
| MID | Total | 30.0 | 30.8 | 32.2 | -7.2% |  |
|  | Unbound | 2.82 | 2.74 | 2.76 | 2.1% | -1% |
|  | % Unbound | 9.4% | 8.9% | 8.6% |  |  |
| HIGH | Total | 129 | 131 | 124 | 4.4% |  |
|  | Unbound | 23.3 | 23.2 | 23.7 | -1.9% | -2% |
|  | % Unbound | 18.0% | 17.8% | 19.2% |  |  |

*^1^Buscher B, Laakso S, Mascher H, Pusecker K, Doig M, Dillen L, et al. Bioanalysis for plasma protein binding studies in drug discovery and drug development: views and recommendations of the European Bioanalysis Forum. Bioanalysis. 2014;6(5):673-82. doi: 10.4155/bio.13.338.*

** Below the LLOQ, but measurable, not used in statistics*

Results and conclusions: Acceptance criteria were met. ETP was stable at 37 °C across all QC levels after 30 minutes of incubation. However, buffered LLOW QCs suffered a loss of 27% after 60 minutes of incubation. % Unbound ETP concentrations were broadly similar (differences ranged between ‑3% and 7%) across all QC levels and incubation times. Hence, 30 minutes was chosen as the incubation time prior to ultrafiltration. Unbuffered samples showed lower free drug concentrations at all QC levels and incubation times, thus demonstrating the importance of buffering samples prior to ultrafiltration.

5.0 Flucloxacillin bioanalysis

5.1 Chemicals and standards

Potassium phosphate, monobasic and dibasic, of Reagent Plus® grade and LCMS grade formic acid was obtained from Sigma-Aldrich. LC/MS-grade acetonitrile and water was purchased from Fisher Scientific (Loughborough, UK). Fresh frozen serum was purchased from TCS biosciences (Botolph Claydon, UK). Flucloxacillin (FLX) was purchased from Sigma Aldrich, and Oxacillin (OFX) (used as an internal standard) from ALSACHIM.

5.2 HPLC-MS/MS conditions and procedure

A Shimadzu Prominence® (Kyoto, Japan) LC system was coupled to a Sciex triple quadrupole 4000 QTRAP mass spectrometer (AB Sciex, Warrington, UK). Sciex Analyst v.1.5.1 software was used to control the HPLC-MS/MS system and collect the MS data. High purity nitrogen was supplied to the MS by a Thyster-TF/1 generator (F-DGSi, Every, France). The LC system consisted of a SIL20AC autosampler, an Agilent G1311a quaternary pump, a CTO-20 AC column oven and a CBM-20A controller. The autosampler was set a temperature of 4 °C and programmed to inject 2 μL of each sample. The mobile phase consisted of 0.1% formic acid in water (solution A) and 0.1% formic acid in acetonitrile (solution B). A constant flow rate of 0.5 mL/min was used and a step-wise gradient elution was started with 98% eluent A for 0.1 min, followed immediately by 40% eluent B for 0.5 min, then increasing to 95% eluent B for 0.5 to 1.0 min, holding at 95% eluent B between 1.0 and 1.5 mins then re-equilibrating at 98% eluent between 1.6 and 4.0 mins. Separation was achieved on a 2.6 µm Kinetex™ XBC18 column (50 x 2.1mm ID) purchased from Phenomenex (Macclesfield, UK). The column was protected by an Upchurch filter unit (Hichrom, Reading, UK) fitted with a 0.5 µm fit. The column oven temperature was set at 40°C. Multiple Reaction Monitoring (MRM) data were collected by the MS using a Turbo V™ source in electrospray ionisation (ESI) positive mode. The source temperature was set at 650 °C, ion spray voltage at 5500 V, ion source gases 1 and 2 at 30 and 40, curtain gas (CUR) at 40 and collision gas (CAD) at 7. Dwell times were set to 20ms. The specific MRM parameters for the analyte and the IS are given below:

| Analyte | Type | Q1 | Q3 | Collision Energy (CE) |
| --- | --- | --- | --- | --- |
| FLX | Quantification | 454 | 295 | 26 |
|  | Qualification |  | 160 | 25 |
| OFX | Quantification | 402.2 | 243 | 18 |
|  | Qualification |  | 144 | 30 |

5.3 Sample preparation and processing

Before injection into the HPLC system, 20μL of sample was dispensed into a 0.5 mL polypropylene Eppendorf® tube. The sample was then mixed thoroughly with 20μL of water and 40μL of OFX (1mg/L) spiked acetonitrile subsequently added and mixed. Patient samples were thawed and vortexed shortly before analysis and processed in the same manner. After being left to stand at ca. 4 °C for 10 min, the sample was centrifuged for 10 min at 17,000 g. 50μL of supernatant was then transferred to a glass HPLC vial containing 450μL of water. The final extract was thoroughly mixed prior to analysis. Then, 2µL of this sample was injected and quantified as described in Section 5.2.

5.4 Plasma protein binding

10 µL of 3M pH 7.4 buffer potassium phosphate buffer was added to 320 µL of serum sample to give a buffer concentration of ca. 0.1M at a pH of 7.40. The buffered sample was incubated at 37°C for 30 minutes and the total FLX concentration quantified as described in sections 5.2 and 5.3.

Unbound FLX was measured by pipetting 250 µL of the serum to be analysed into a Centrifree® Ultrafiltration Device (Merck Millipore, Watford, UK). After centrifugation (1500 g, 20 min, 37°C), 20 μL of each filtrate was dispensed into a 0.5 mL polypropylene Eppendorf® tube and 20 μL of blank serum added, followed by mixing. This sample was then extracted and analysed as described in sections 5.2 and 5.3 FLX concentrations were calculated using calibrators prepared in serum.

6.0 Linezolid bioanalysis

6.1 Chemicals and standards

Heptane Sulphonic Acid and Sodium Hydroxide (NaOH) was obtained from Sigma-Aldrich. Ortho Phosphoric Acid (OPA) was obtained from VWR. HPLC grade methanol (MeOH), acetonitrile (MeCN) and water were purchased from Fisher Scientific (Loughborough, UK). Fresh frozen serum was purchased from TCS biosciences (Botolph Claydon, UK). Linezolid (LZD) was purchased from Sigma Aldrich.

6.2 HPLC-MS/MS conditions and procedure

A Gilson 307 pump was coupled to a Dionex Gina 50 autosampler and 3000VWD/3400 UV detector. Dionex Chromeleon v.6.8 software was used to control the HPLC-UV system and collect the UV data. The autosampler was programmed to inject 10 μL of each sample.

The mobile phase consisted of 500mg/L of Heptane sulphonic acid in 69:30:1 H2O/MeOH/OPA (v/v/v) respectively, pH adjusted to 5.0 using NaOH. Flow rate of 1.5 mL/min was using isocratic elution. Separation was achieved on a 5.0 µm Hypersil™ ODS column (50 x 2.1mm) purchased from Thermo Scientific. The detection wavelength was set to 254nm.

6.3 Sample preparation and processing

Before injection into the HPLC system, 100μL of sample was dispensed into a 0.5 mL polypropylene Eppendorf® tube. The sample was then mixed thoroughly with 100μL acetonitrile. Patient samples were thawed and vortexed shortly before analysis and processed in the same manner. After being left to stand at ca. 4 °C for 5 min, the sample was centrifuged for 5 min at 17,000 g. 100μL of supernatant was then transferred to a glass vial and thoroughly mixed prior to analysis. Then, 10µL of this sample was injected and quantified as described in Section 6.2.

The analytical range of this assay is 0.5-40mg/L.

7.0 Teicoplanin bioanalysis

Teicoplanin is analysed by QMS based immunoassay run on the Indiko Plus® (Thermo Scientific). The analytical range for this assay is 5.0-50mg/L.

**Table S1. BSAC OPAT treatment aims^(1)^**

| Cure | To complete an agreed OPAT duration of therapy on either intravenous and/or complicated oral antimicrobials with no requirement for long-term antimicrobial therapy. |
| --- | --- |
| Improvement | To complete an agreed OPAT duration of therapy on either intravenous and/or complicated oral antimicrobials   1. as part of an agreed surgical infection management plan with further surgery planned, or 2. where there is a requirement for subsequent long-term or an extended course of oral suppressive antimicrobial therapy, or 3. where potentially infective prosthetic material is still in situ. |
| Palliation | To undertake a course of OPAT on either intravenous and/or complicated oral antimicrobials where there are agreed ceilings of care due to comorbidities, with death being the likely outcome. |

**Table S2. BSAC OPAT outcomes^(1)^**

| Attained-uncomplicated | - No unplanned changes in antimicrobial agent. - No adverse events. - No planned or unplanned readmission related to the current OPAT episode. - No readmission of ≥24h for unrelated event (i.e. daycase/overnight stayf or another medical problem allowed). |
| --- | --- |
| Treatment aim attained-complicated | Completed OPAT therapy as per treatment aim but with one or more of the following:   - Unplanned changes in antimicrobial agent. - Any adverse event including readmission for <24h related to the current OPAT episode. |
| Treatment aim not attained | - Failure to complete planned OPAT therapy for any reason other than readmission due to unrelated event - Worsening of infection requiring readmission. - Readmission for ≥24h for any cause related to OPAT, including adverse events. |
| Indeterminate | Readmission for ≥24 h due to unrelated event. |
| Death | Death due to any cause, except palliation. |

**References**

1. Chapman ALN, Patel S, Horner C, Gilchrist M, Seaton RA. Outpatient parenteral antimicrobial therapy: updated recommendations from the UK. *J Antimicrob Chemother* 2019; **74**: 3125–3127.

**Table S3. Adverse Events**

| Drug | n | Neutropenia | Thrombocytopenia | ALT>ULN | ALT>3xULN | ALP>ULN | ALP>3xULN |
| --- | --- | --- | --- | --- | --- | --- | --- |
| Ceftriaxone | 20 | 5 | 0 | 8 | 0 | 2 | 1 |
| Ceftriaxone and daptomycin | 1 | 0 | 0 | 0 | 0 | 0 | 0 |
| Ceftazidime | 3 | 1 | 0 | 2 | 0 | 0 | 0 |
| Daptomycin | 1 | 0 | 1 | 0 | 0 | 1 | 0 |
| Ertapenem | 5 | 0 | 0 | 0 | 0 | 2 | 0 |
| Ertapenem and daptomycin | 1 | 1 | 0 | 1 | 0 | 1 | 0 |
| Flucloxacillin | 3 | 1 | 1 | 0 | 0 | 1 | 0 |
| Linezolid | 1 | 0 | 0 | 0 | 0 | 0 | 0 |
| Teicoplanin | 4 | 2 | 2 | 1 | 0 | 1 | 0 |

ALT ULN = 34 IU/L, ALP ULN = 130 IU/L, neutropenia defined as neutrophils <2 x10^9^/L, thrombocytopenia defined as platelets <135 x10^9^/L. ALT, alanine transaminase; ALP, alkaline phosphatase; ULN, upper limit of normal.
